# Artificial intelligence in paediatric chest imaging: applications, challenges, and future directions

**DOI:** 10.1007/s00247-026-06671-6

**Published:** 2026-06-03

**Authors:** John Joseph Muringathuparambil, Shamiek Maharaj, Bradley Max Segal, Nasreen Mahomed

**Affiliations:** Faculty of Health Sciences, University of the Witwatersrand, 7 York Road, Parktown, Johannesburg, 2193 South Africa

**Keywords:** Artificial intelligence, Child, Diagnostic imaging, Low- and middle-income countries, Lung diseases, Radiography, thoracic, Tomography, X-ray computed, Ultrasonography

## Abstract

**Background:**

Paediatric chest imaging is central to diagnosing respiratory and cardiopulmonary disease, particularly in low- and middle-income countries (LMICs) where pneumonia remains a leading cause of childhood mortality and radiology expertise is scarce. Artificial intelligence (AI) could expand access, standardise quality and support task-shifting in these “diagnostic deserts,” yet most systems are trained and validated on adult datasets from high-income settings, and paediatric radiographs form only a small minority of major public training cohorts — raising concerns about safety, generalisability and equity when such models are deployed in children.

**Objective:**

To synthesise current applications of AI across paediatric chest radiography, lung ultrasound, computed tomography and MRI, with emphasis on LMIC workflows, and to define what is required for safe, paediatric-specific deployment.

**Materials and methods:**

Narrative/pictorial review of the published literature, complemented by the authors’ real-world evaluation of a generalist vision–language model (MedGemma) on adult and paediatric chest radiographs, illustrated with representative clinical cases.

**Results:**

Beyond diagnosis, AI shows potential in quality assurance, lung-ultrasound guidance and multilingual reporting. Real-world experience from CAD4Kids and from MedGemma’s evaluation — including critical failures in detecting pericardial effusion and tuberculosis without explicit clinical context — illustrates common failure modes and the ethical implications of domain shift. Key challenges include infrastructure constraints, dataset scarcity and the need for age-aware, explainable models.

**Conclusion:**

Rather than adapting adult systems, future tools must be designed from inception for children and the environments in which they live, prioritising federated learning, multimodal integration, robust validation across age strata and multilingual communication with caregivers.

**Graphical abstract:**

**Figure:**
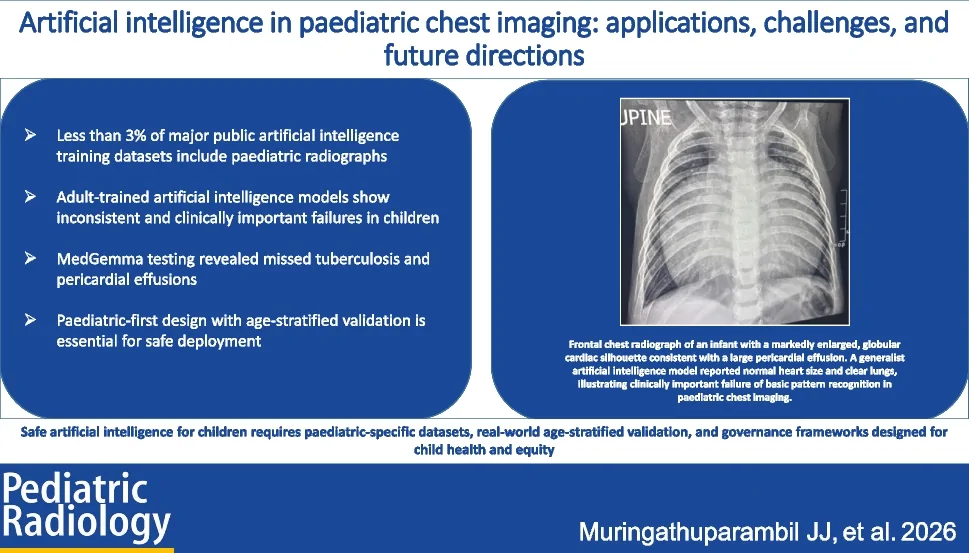

**Supplementary information:**

The online version contains supplementary material available at https://doi.org/10.1007/s00247-026-06671-6.

## Introduction and clinical context

### Global burden and the paediatric imaging gap

Paediatric chest imaging is a core diagnostic tool for respiratory infections, congenital abnormalities, and cardiopulmonary disease across diverse health systems. Pneumonia remains a leading infectious cause of death in children under five, with the burden disproportionately concentrated in low- and middle-income countries (LMICs) [1]. This clinical burden sits alongside structural constraints: radiologist shortages in many regions and limited paediatric subspecialty coverage, even where imaging volume is high.

Paediatric interpretation is not simply “adult radiology in miniature”. Age-dependent anatomy, evolving physiology and normal developmental variants introduce recurring pitfalls for non-paediatric readers [2, 3]. Image acquisition is often challenging—motion, rotation and suboptimal inspiration are common—whilst radiation sensitivity narrows the margin for repeat imaging. These constraints make diagnostic support attractive, but they also heighten the consequences of error.

Artificial intelligence (AI) therefore presents both opportunity and risk. In principle, AI could support task-shifting, improve consistency and strengthen workflows in resource-constrained settings. In practice, many systems have been trained and evaluated predominantly on adult data acquired in high-resource environments, with limited representation of paediatric imaging features or LMIC acquisition variability. This mismatch motivates the central question of this review: what is currently feasible, what fails in real-world paediatric settings and what would be required for safe integration.

### The validation gap: when adult artificial intelligence meets paediatric reality

**Fig. 1:**
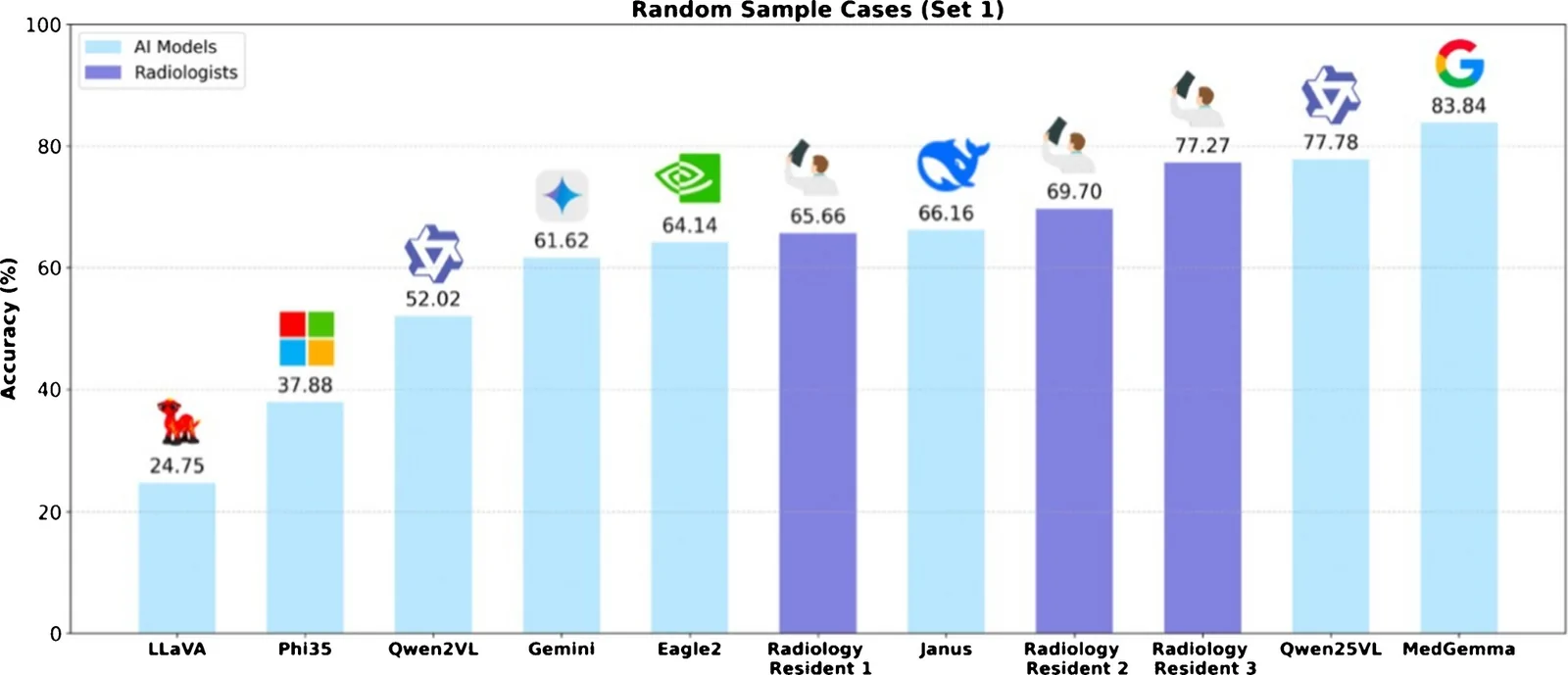
ReXVQA reader-study performance of MedGemma (4B) compared with the three radiology residents in that benchmark [4]. On the 200 randomly sampled cases shown, MedGemma achieved 83.84% accuracy versus 77.27% for the best-performing resident. This result should not be interpreted as superiority over subspecialist thoracic radiologists or as evidence of paediatric clinical readiness

A central barrier to paediatric AI adoption is the validation gap created by adult-dominant training and evaluation. Major public datasets include only a small proportion of paediatric images, limiting the reliability of performance claims in children [3]. Benchmarks may nevertheless appear compelling. MedGemma (4B multimodal variant; developmental at time of writing) achieved the highest overall accuracy among the eight models evaluated on the ReXVQA chest-radiograph interpretation benchmark (83.24%); on the accompanying 200-case reader study it scored 83.84%, exceeding the best-performing radiology resident (77.27%) (Fig. 1) [4]. In ReXVQA, the comparison readers were radiology residents rather than subspecialist thoracic radiologists, so “expert” performance cannot be inferred from that benchmark alone. In report-generation evaluations, board-certified radiologist review has also been used, highlighting that “good” performance depends strongly on task definition and evaluation design. Where adult-derived evidence is discussed in this review, it should be read as methodological precedent rather than validated paediatric performance, and extrapolation to children requires age-stratified data to support it.

Such results are a signal of technical progress, but they do not establish safety or effectiveness in paediatric populations, nor do they guarantee robustness under acquisition variability and epidemiologic patterns typical of LMIC practice—including settings where tuberculosis is endemic and image quality constraints are common.

Ethical and governance considerations are intensified in paediatrics. Errors can alter life-course outcomes, and children cannot meaningfully consent or advocate for themselves. However, the generalisation problem is not reducible to geography alone: models can fail when transferred across contexts that differ in disease prevalence, comorbidity patterns, equipment and protocols, workforce skill mix and temporal shifts in practice and population health [5]. For these reasons, paediatric AI should be assessed using context-specific validation and monitored performance in the environments where children are imaged, rather than relying on adult-dominant benchmarks as a proxy for clinical readiness.

### Low- and middle-income countries realities and innovation imperatives

LMIC deployment barriers extend beyond workforce limitations to infrastructure constraints that shape both image acquisition and AI implementation. Analogue equipment persists in some settings; PACS integration and stable connectivity may be limited; and imaging may be acquired by non-specialist staff and interpreted by general clinicians, if interpretation occurs at all. These “diagnostic deserts” are precisely where AI could provide disproportionate benefit—if tools are engineered for portability, low-bandwidth or offline operation and resilience to interruptions in power and workflow.

The CAD4Kids initiative illustrates both promise and the need for local calibration. Developed through collaboration between South African clinicians and Dutch engineers, CAD4Kids achieved 76% sensitivity and 80% specificity for WHO-defined pneumonia detection at a large tertiary hospital serving children in an LMIC context [6]. Importantly, performance varied by subgroup (including HIV exposure status and demographic factors), emphasising that paediatric AI tools require population-aware validation and ongoing monitoring rather than assuming uniform performance across settings.

### Beyond diagnosis: expanding artificial intelligence applications

Although most clinical AI discussions centre on pathology detection, there are adjacent applications that may meaningfully improve paediatric care in LMIC contexts. Natural language processing (NLP) systems could support translation of radiology outputs into local languages or generate caregiver-facing explanations, including voice-based communication, where health literacy and language barriers affect comprehension and adherence.

AI-enabled quality assurance tools may also assist at the point of acquisition. Real-time detection of motion or positioning issues could prompt corrective action before poor-quality studies enter the reporting pathway. Standardised acquisition guidance may improve consistency in environments where paediatric-specific training is limited.

Taken together, these considerations argue for paediatric-first development: globally inclusive datasets, explicit context-specific validation and deployment designs that reflect real LMIC workflow constraints and the ethical stakes of child health. The goal should not be to transplant adult systems into paediatrics, but to build approaches that begin with paediatric anatomy, disease patterns and care pathways as the design baseline.

## Current applications and real-world performance

### Chest radiography: promise meets practical limitations

Chest radiography remains the backbone of paediatric thoracic imaging and, in many low- and middle-income countries (LMICs), the only modality consistently available. This reality explains why thoracic AI development has been heavily centred on chest radiographs. Many landmark models trained on large adult datasets report strong performance for pneumonia, pleural effusion and cardiomegaly detection [7–11]; however, these results derive primarily from adult cohorts and should be interpreted as methodological precedents rather than validated paediatric solutions. Paediatric images constitute only a small minority of major public datasets (Table 1), and external validation studies repeatedly show that adult-trained systems may generalise poorly to children [2].

**Table 1:** Overview of selected publicly available datasets used in paediatric chest imaging artificial intelligence (AI) research

Dataset	Total images	Paediatric %	Primary use case
NIH Chest X-ray14	~112,000	<1%	Adult pathology detection
CheXpert	~224,000	<2%	Multi-label classification
MIMIC-CXR	~377,000	<1%	Report generation
PadChest	~160,000	~3%	Mixed pathology
Guangzhou Pediatric	5,856	100%	Paediatric pneumonia
CAD4Kids	1,000+	100%	LMIC paediatric TB/pneumonia

Dataset characteristics compiled from systematic review findings [3] and original dataset publications [8–10]. CAD4Kids performance and cohort characteristics are reported in [6]. The Guangzhou paediatric pneumonia dataset is described in [11]. PadChest cohort statistics are summarised in [3]. Analysis based on publicly reported statistics and research literature

*LMIC* low- and middle-income country, *TB* tuberculosis

**Fig. 2:**
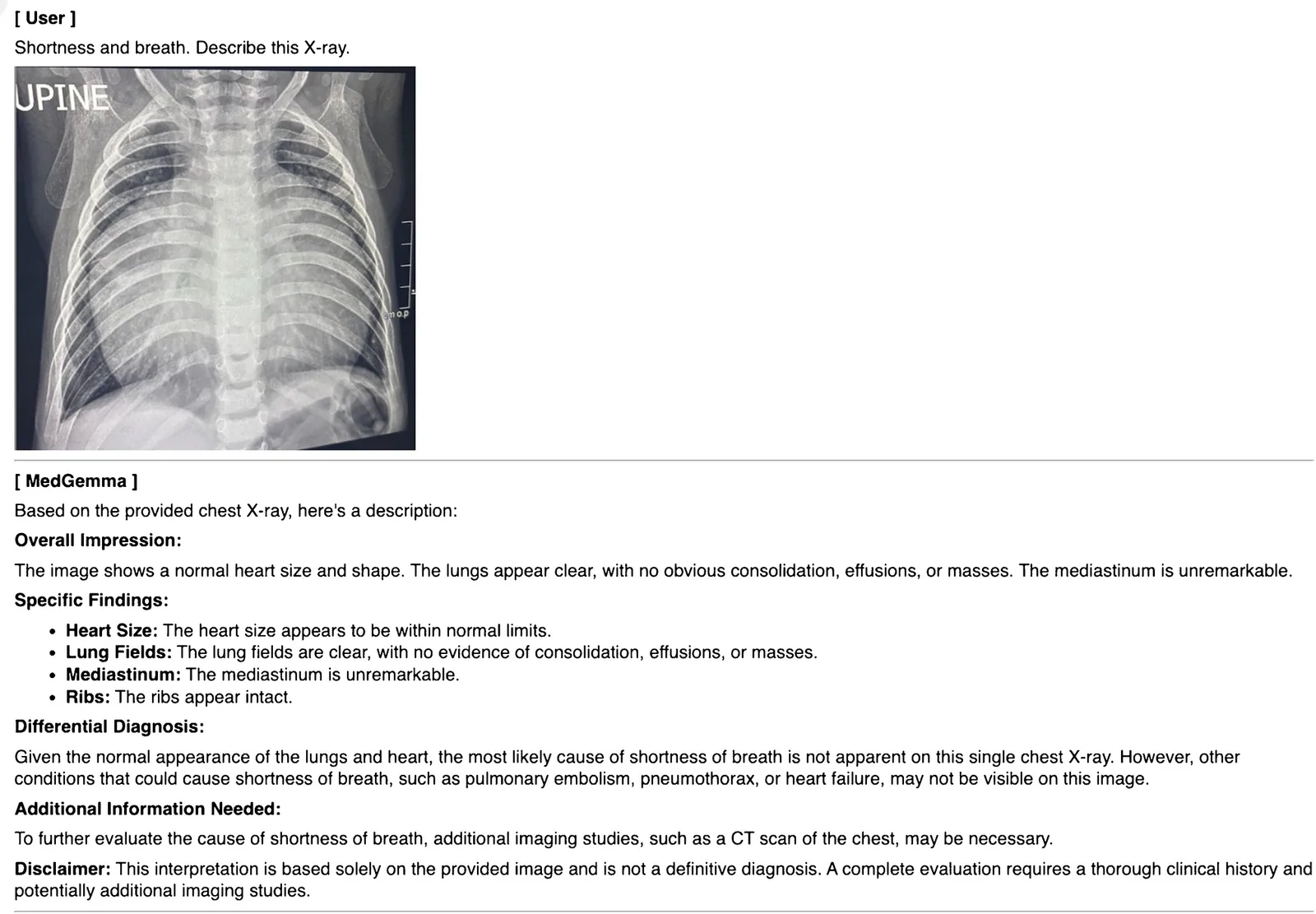
A young male infant with a large pericardial effusion. Frontal chest radiograph demonstrates a markedly enlarged, globular cardiac silhouette consistent with significant pericardial effusion; MedGemma 4B-it reported normal heart size and clear lungs, illustrating clinically important failure of basic pattern recognition under domain shift and paediatric acquisition variability

Our evaluation of a contemporary vision–language model during an African health-AI exercise illustrates both capability and fragility in real clinical use. The model performed reliably on clear adult-pattern pneumonias and reproduced its previously reported benchmark performance on the ReXVQA dataset (Supplementary Material 1; see Section 1 for benchmark context). When applied to paediatric clinical radiographs from an LMIC setting, outputs became more variable and sensitive to acquisition conditions and local disease context. In a confirmed paediatric pulmonary tuberculosis case, the model initially described a non-specific “left upper lobe opacity” without identifying cavitation or suggesting tuberculosis (Supplementary Material 2). In settings where tuberculosis is endemic, this is a clinically important limitation because TB recognition is one of the few high-yield reasons chest radiographs remain routinely used in many paediatric respiratory pathways. In a second case, the model identified an oesophageal foreign body on an upright frontal radiograph but failed to detect the identical object on a rotated, supine projection (Supplementary Material 3). More concerning, the generated output in this case also contained implausible additional findings and diagnoses not supported by the image - an example of confabulation risk when generative systems are used outside tightly constrained tasks. This behaviour is not a cosmetic limitation: in paediatrics, confidently stated but incorrect “report-like” outputs can misdirect escalation decisions, antibiotic use, referral pathways and caregiver counselling unless the model is tightly constrained to validate tasks with explicit uncertainty signalling. In a separate evaluation on an adult case from a high-burden setting, the model similarly returned broad differentials without explicitly naming tuberculosis despite classic radiographic features (Supplementary Material 4). In a third case, the model missed a pericardial effusion despite a globular cardiac silhouette (Fig. 2).

These cases highlight recurring limitations of current paediatric CXR AI and multi-modal generative models: (1) limited paediatric representation in training data, (2) vulnerability to projectional and technical variation typical of paediatric imaging and (3) incomplete modelling of locally prevalent conditions. They also underscore a safety-relevant point: fluent “explanations” can obscure uncertainty and may introduce fabricated diagnoses unless systems are bounded by validated outputs, calibrated confidence and appropriate human oversight.

Near-term clinical value is therefore most plausible in narrow, constrained applications—line and tube localisation, automated quality assessment, prioritisation of grossly abnormal studies and decision support for non-specialist clinicians—provided these tools undergo explicit paediatric-specific and LMIC-relevant validation and are evaluated for failure modes, including confabulation. Ultimately, progress in this domain depends on child-specific dataset development, real-world benchmarking and evaluation within the health systems where these tools are intended to function.

### Lung ultrasound at the point of care: reducing operator dependence

AI development in paediatric chest imaging has skewed toward chest radiography largely because adult CXR datasets are abundant, not because CXR is always the optimal modality in children. For several common paediatric indications, lung ultrasound (LUS) performs well and avoids ionising radiation [12, 13]. The practical limitation of LUS is not its diagnostic potential but its operator dependence—an area where AI may offer meaningful support [14].

Two directions are particularly relevant. First, AI-assisted acquisition guidance can provide real-time feedback to help less-experienced users obtain diagnostically adequate images. A multi-centre study demonstrated that healthcare professionals without prior ultrasound experience, when assisted by an AI guidance tool, could acquire scans of diagnostic quality comparable to expert-acquired studies [14]. Second, automated interpretation models have been developed to detect and quantify key sonographic features such as consolidation, B-lines and pleural effusions, with the goal of improving consistency at the bedside [15, 16]. Whilst early results are encouraging, paediatric evidence remains limited relative to adult literature, and performance is likely to be sensitive to device type, operator experience, scanning protocol and case mix - therefore multi-centre paediatric validation remains a prerequisite for routine deployment.

If these tools remain robust outside controlled development settings, they could support task-shifting in contexts where expert sonographers and radiologists are scarce, enabling earlier diagnosis and triage for high-burden diseases such as pneumonia. However, real-world paediatric deployment depends on careful validation across ages and clinical environments, and on ensuring that AI outputs integrate safely into clinical decision-making rather than replacing training and supervision [17, 18].

### Computed tomography: evolving capability and persistent limitations

Computed tomography (CT) provides detailed structural assessment of the paediatric thorax, including congenital anomalies, complications of pneumonia (e.g. necrotising infection/empyema), airway disease and oncological staging. Access remains uneven globally, and radiation exposure requires careful paediatric dose optimisation. Although AI research in chest CT is expanding, paediatric-specific evidence remains limited for several common paediatric indications—including congenital lung malformations and other routine paediatric CT referral questions—reflecting the broader scarcity of paediatric CT datasets.

Drawing primarily on adult data, AI advances in CT have been particularly visible in reconstruction and image optimisation—supporting low-dose imaging and potentially improving quality where motion and noise degrade interpretability. Emerging techniques such as denoising and motion correction may further improve image quality at lower dose, whilst synthetic augmentation approaches may help address data scarcity [19–22]. Nevertheless, paediatric-specific CT datasets remain relatively limited, and heterogeneity in acquisition parameters and age-related anatomy can constrain generalisability. For this reason, adult-derived performance claims should not be assumed to translate directly to paediatric practice. Multi-centre paediatric validation remains essential before broad clinical adoption.

### Magnetic resonance imaging: precision, motion, complexity and the promise of acceleration

Magnetic resonance imaging (MRI) is increasingly used in paediatric thoracic assessment for mediastinal masses, congenital structural abnormalities, cardiac evaluation and chest wall pathology. Its advantages are offset by longer acquisition times, motion artefact and the potential need for sedation in younger children. Developed largely in adult populations, AI-enabled acceleration, reconstruction and motion-correction techniques have shown promise in reducing scan times and improving signal-to-noise ratios [23–26]. Although many of these approaches were developed in adult neuro-oncology and cardiac imaging, their technical value is directly relevant to paediatric thoracic MRI.

Federated learning may help address the chronic shortage of paediatric MRI datasets by enabling multi-institutional training without centralising raw patient data [27–29]. In parallel, radiomics and longitudinal modelling are being explored for risk stratification and outcome prediction in paediatric oncology [30–32]. As with CT and radiography, protocol heterogeneity, age variation and limited ground-truth standards remain barriers, reinforcing the need for deliberate paediatric-specific validation prior to routine clinical deployment.

## Technical and deployment challenges

### The infrastructure reality

Real-world deployment of AI in paediatric imaging faces constraints that laboratory benchmarks rarely capture. Experience within a large public healthcare system in a low- and middle-income country (LMIC) illustrates how basic infrastructure limitations—intermittent connectivity, ageing PACS hardware, limited storage and inconsistent integration between systems—can negate the practical value of otherwise capable algorithms. This is the central implementation paradox: the diagnostic gap AI could help address is often greatest in precisely the settings where deployment is hardest.

Children add a second layer of complexity at the point of acquisition. Motion artefact, rotation, variable inspiration and age-dependent anatomy are common in paediatric chest radiographs and can destabilise adult-trained systems. In our limited evaluation of MedGemma 4B-it during the AfriDSAI Health AI Datathon (Fig. 2), even modest projectional variation was associated with confident but incorrect outputs, including clinically important misses—an operational risk in busy services where downstream teams may over-trust fluent model responses [33].

### Paediatric generalisability: data scarcity, domain shift and failure modes

The gap between benchmark performance and clinical utility becomes most visible when models encounter paediatric normal variants and paediatric-specific context. In one infant case (Fig. 2), the model did not identify a large pericardial effusion despite a markedly enlarged, globular cardiac silhouette. This should not be framed as a “paediatric-only” pathology problem; rather, it illustrates a more general failure mode of brittle pattern recognition under domain shift, where model confidence exceeds competence.

This is not limited to isolated examples. The thymus, age-dependent cardiothoracic ratios and developmental variation in lung appearance create interpretive contexts that adult-dominant training distributions may mishandle [2, 3]. In children, these errors have outsized consequences: false reassurance can delay escalation, whilst over-calling normal variants can trigger unnecessary antibiotics, repeat imaging, radiation exposure or referral. For paediatric deployment, the core question is therefore not whether a system can score well on a benchmark, but whether it can remain reliable across the real-world variability that defines paediatric imaging services—and whether its uncertainty is communicated in a way that discourages over-trust.

### Workflow integration and governance are safety problems, not software features

Even accurate algorithms can fail clinically if they sit outside routine workflows. Safe implementation requires integration into worklists and PACS viewers, transparent display of uncertainty, clear logging of model outputs and mechanisms for clinician override and feedback. “Invisible co-pilot” metaphors are appealing, but in paediatrics they can obscure accountability: who is responsible when an algorithm influences a missed diagnosis, delayed treatment or unnecessary intervention? Deployment should instead prioritise explicit audit trails, calibrated uncertainty display and clear responsibility pathways when outputs influence decisions.

Accordingly, implementation should be treated as a governance problem. Health systems require auditable outputs, version control, post-deployment monitoring for drift and subgroup underperformance and defined escalation pathways when model behaviour deviates from expectations. These requirements are amplified in LMIC settings due to older vendor software, limited interoperability and constrained IT support. Hybrid deployment strategies are therefore often more realistic than cloud-only designs. Edge-capable systems that can function offline—with periodic synchronisation when connectivity permits—better match many public-sector environments. The technical target in these settings is not maximal performance under ideal conditions, but predictable performance, graceful degradation when systems fail and a deployment pathway that can be supported long-term.

Accountability and liability must be defined before deployment. If an AI output contributes to harm (through missed diagnosis, biased outputs or fabricated “findings”), responsibility cannot be ambiguous. Implementation policies should specify: (i) whether AI outputs enter the medical record and how they are labelled, (ii) who is accountable for AI-influenced decisions (clinician, institution, developer or shared), (iii) how version changes are governed and audited and (iv) how privacy, cybersecurity and access controls are enforced—particularly where systems operate offline or on edge devices in LMIC settings.

### Toward paediatric-centric design: robustness, calibration and communication

Paediatric imaging AI should be designed around three priorities: robustness, calibration and communication. Robustness means resilience to paediatric acquisition issues (rotation, motion, low inspiration) and device/protocol heterogeneity. Calibration means that model confidence tracks true reliability, enabling clinicians to recognise when outputs should be discounted. Communication means outputs that can be interpreted across users (radiologists, general clinicians and—in selected applications—care teams), including explicit uncertainty indicators and, where appropriate, multilingual or caregiver-facing explanations.

Crucially, “acceptable performance” cannot be defended using a single headline accuracy figure. Thresholds must be task-specific and harm-aware: triage and quality assurance tools carry different risk than systems that influence treatment decisions. For this reason, paediatric deployment should be tied to prospective evaluation stratified by paediatric age bands, with monitoring frameworks that detect drift, failure clusters and subgroup underperformance over time.

The trajectory of models such as MedGemma—showing strong performance compared with radiology resident readers on standardised benchmarks—does not eliminate the paediatric validation problem; it sharpens it [4]. The urgent work is to define safe tasks, validate them in paediatric cohorts under real acquisition conditions and embed them into workflows with auditable outputs and clear accountability.

## The technological roadmap to paediatric-centric artificial intelligence

Having established why adult-centric performance does not automatically translate to paediatrics—particularly in resource-constrained settings—the next step is a pragmatic roadmap. The goal is not simply “more paediatric data”, but deliberate choices about what tasks to automate, how models learn from scarce data and how outputs are governed in clinical care.

### Quantitative biomarkers and opportunistic screening

The future of paediatric AI extends beyond detecting overt pneumonia or effusion. One opportunity is extracting quantitative, often sub-visual biomarkers from routine chest radiographs and converting them into reproducible longitudinal measures [34–37]. For chronic disease such as cystic fibrosis, models may generate severity scores over time that support monitoring and stratification, potentially reducing reliance on repeated CT when appropriate and validated. Adult work suggests that subtle opacity and interstitial change may be detectable beyond human sensitivity; paediatric translation would require age-specific normative standards and careful validation before clinical use [36, 37].

Opportunistic screening is a related direction. Radiographs obtained for respiratory symptoms could be automatically assessed for skeletal demineralisation patterns or body-composition proxies relevant to nutrition and cardiometabolic risk, analogous to adult work using chest imaging for osteoporosis risk stratification and body-composition assessment [38–41]. In paediatrics, any such approach would require explicit governance: age-appropriate reference ranges, defined care pathways for incidental findings and safeguards against inequitable downstream consequences.

### Multimodal integration and predictive analytics

AI performance can improve when imaging is interpreted with clinical context rather than in isolation. Transformer-based architectures can integrate radiographs with structured clinical variables and unstructured notes [42]. This directly relates to observed failure modes in our own testing, where provision of clinical context altered outputs; mature systems should ingest such context automatically rather than relying on ad hoc prompting [42].

Longitudinal modelling extends the aim from diagnosis to prediction. Adult studies show that combining radiographs with early electronic health record data can forecast deterioration or ICU transfer with clinically useful accuracy [43]. In paediatric emergency and critical-care settings, analogous models could support earlier escalation triggers—provided they are validated prospectively and assessed for subgroup performance and unintended consequences.

### Learning from scarce, distributed paediatric data

Paediatric imaging is data-sparse by nature: rarer diseases, smaller volumes, fragmented systems. Few-shot and meta-learning techniques aim to learn meaningful patterns from limited labelled examples [44]. Self-supervised learning using large volumes of unlabelled paediatric radiographs can provide robust representations of anatomy and normal variants before task-specific fine-tuning [45], reducing dependence on heavily curated labels.

Federated learning offers a complementary path where privacy and data governance prevent centralisation. Hospitals can collaboratively train shared models whilst keeping images behind local firewalls, exchanging only model updates [28, 29]. This is particularly relevant for building globally representative paediatric tools without excluding LMIC institutions that cannot export data.

### Explainable and age-aware artificial intelligence

Safe adoption depends on systems that are interpretable and explicitly age-aware. Explainability tools (e.g. saliency methods) can reveal where a model is “looking”, sometimes helping radiologists detect obvious failure modes [46]. However, plausible-looking explanations can also worsen automation bias if clinicians over-trust convincing but incorrect rationales. Explanations therefore require validation, and radiologists need training to interpret them critically rather than as proof [47].

Age-aware evaluation is non-negotiable. Lung size, thymic contour, cardiothoracic ratio and ossification patterns evolve dramatically from neonate to adolescent. Adult-trained models may misclassify normal paediatric variants as pathology [2, 3]. Safer design strategies include using age or developmental stage as explicit inputs, training age-stratified models and reporting performance separately across predefined paediatric age bands with prospective validation thresholds.

### From assistant to more agentic workflows

In the near term, AI is most plausibly deployed as constrained workflow support: prioritising worklists, flagging critical or discrepant studies, performing routine measurements and suggesting structured report elements whilst surfacing relevant clinical context [50]. This can reduce cognitive load and variability whilst keeping radiologists responsible for final interpretation.

Longer-term research is exploring more agentic systems capable of executing multi-step workflows (e.g. detecting a critical finding, cross-checking prior imaging and vitals, drafting a report and issuing a time-stamped alert). Whether such systems are acceptable in paediatric care remains uncertain, but progress in this direction increases the importance of audit trails, data standards, and safety governance—requirements that will benefit any clinical AI deployed in children [48, 49].

## Future directions: building for children, not datasets

### Breaking free from adult-centric development paradigms

Paediatric AI development remains constrained by a common pattern: adult-trained models that are subsequently adapted with relatively small paediatric datasets. This is not only a data limitation; it is a design misalignment. Children differ from adults in anatomy, disease prevalence and the frequency of acquisition artefacts, and these differences shape both model failure modes and clinical risk. An approach designed from inception for children therefore requires deliberate problem selection (which tasks are safe and valuable), age-aware evaluation, and deployment pathways designed for the clinical environments in which children are actually imaged.

The ReXVQA benchmark exemplifies the evolution of visual question answering (VQA) evaluation, but its paediatric representation remains limited, reinforcing an adult-centric bias despite technical sophistication [3]. Addressing this is not simply a call for “more data”. Paediatric imaging data are inherently fragmented across institutions and constrained by governance, consent, and small case volumes. Future progress will depend on multi-institutional collaboration, including privacy-preserving approaches such as federated learning, and on clearly defined paediatric evaluation sets that reflect endemic disease patterns, acquisition variability and age-stratified normal appearances.

Our datathon evaluation of MedGemma was undertaken with explicit awareness of its limitations: an adult-trained generalist model applied to paediatric chest radiographs without domain-specific adaptation and evaluated only in a 4-billion parameter configuration. The failures we observed were therefore not surprising; they illustrate known constraints when general-purpose models are applied outside their training distribution. However, the same evaluation also clarified where near-term value may plausibly exist: narrow, safety-oriented tasks such as foreign body detection, line and tube localisation, and acquisition quality assessment. In under-resourced settings where radiologists are scarce, targeted tools that reduce avoidable harm (missed tubes/lines, delayed detection of gross abnormalities, poor-quality images entering the pathway) may be more realistic and defensible than attempts at broad, autonomous diagnosis.

The key question is therefore not whether AI will be used, but how it will be built, who it will serve, and whether its deployment will be aligned with the needs and constraints of under-resourced health systems. In paediatrics, “technical performance” must be interpreted through a safety lens: error modes, subgroup performance, confidence calibration and the downstream clinical consequences of both misses and false positives. Importantly, current generalist models should be treated as research tools or narrowly scoped assistants, not as near-equivalents of trained paediatric radiologists requiring only “local induction” into a local environment. Their clinical use should therefore be limited to clearly defined tasks with prospective, age-stratified validation, explicit uncertainty display and auditable governance.

### Explainability as communication, not just transparency

Explainability in paediatric AI extends beyond showing “where the model looked”. Clinicians need outputs that support accountable decision-making (structured findings, uncertainty indicators and auditability), whilst caregivers require explanations that are comprehensible, culturally appropriate and available in local languages. Evidence from an international survey of health-care professionals suggests that acceptance of AI in paediatric imaging is strongly influenced by clarity of explanation and visible human oversight [51].

Future systems should therefore support layered communication: technical annotations for radiologists, concise summaries for referring clinicians and simplified or voice-based explanations for families where health literacy and language barriers limit comprehension. Importantly, caregiver-facing tools should be framed as communication support rather than as diagnostic authority, and they require governance to prevent overconfidence, misunderstanding and unintended coercion.

### Human-centred co-design and participatory deployment

A recurring limitation of AI deployment is the assumption that technical success will translate automatically into clinical adoption. In LMIC paediatric imaging, this is rarely true. Co-design—where clinicians, radiographers, caregivers and local healthcare workers participate as collaborators—should be treated as a core development requirement rather than an implementation afterthought. Participatory engagement can shape what is built (high-value tasks), how it is presented (interfaces, language, uncertainty) and how it is governed (escalation pathways, accountability, documentation). This approach also improves the realism of evaluation: models can be tested against the actual failure conditions that matter (poor positioning, intermittent connectivity, non-specialist users) rather than idealised datasets.

Participatory design has already been demonstrated in several paediatric imaging AI initiatives. The CAD4Kids programme, for example, involved active collaboration between South African clinicians, radiographers and Dutch engineers to define clinically meaningful diagnostic targets and calibrate model detection thresholds for local disease patterns, including in a population with high HIV exposure rates [6]. Similarly, AI-assisted lung ultrasound guidance projects have incorporated frontline healthcare workers—including those with no prior ultrasound experience—into iterative development and testing cycles, with the aim of ensuring that acquisition guidance tools reflect real bedside workflows rather than idealised laboratory conditions [14].

**Fig. 3:**
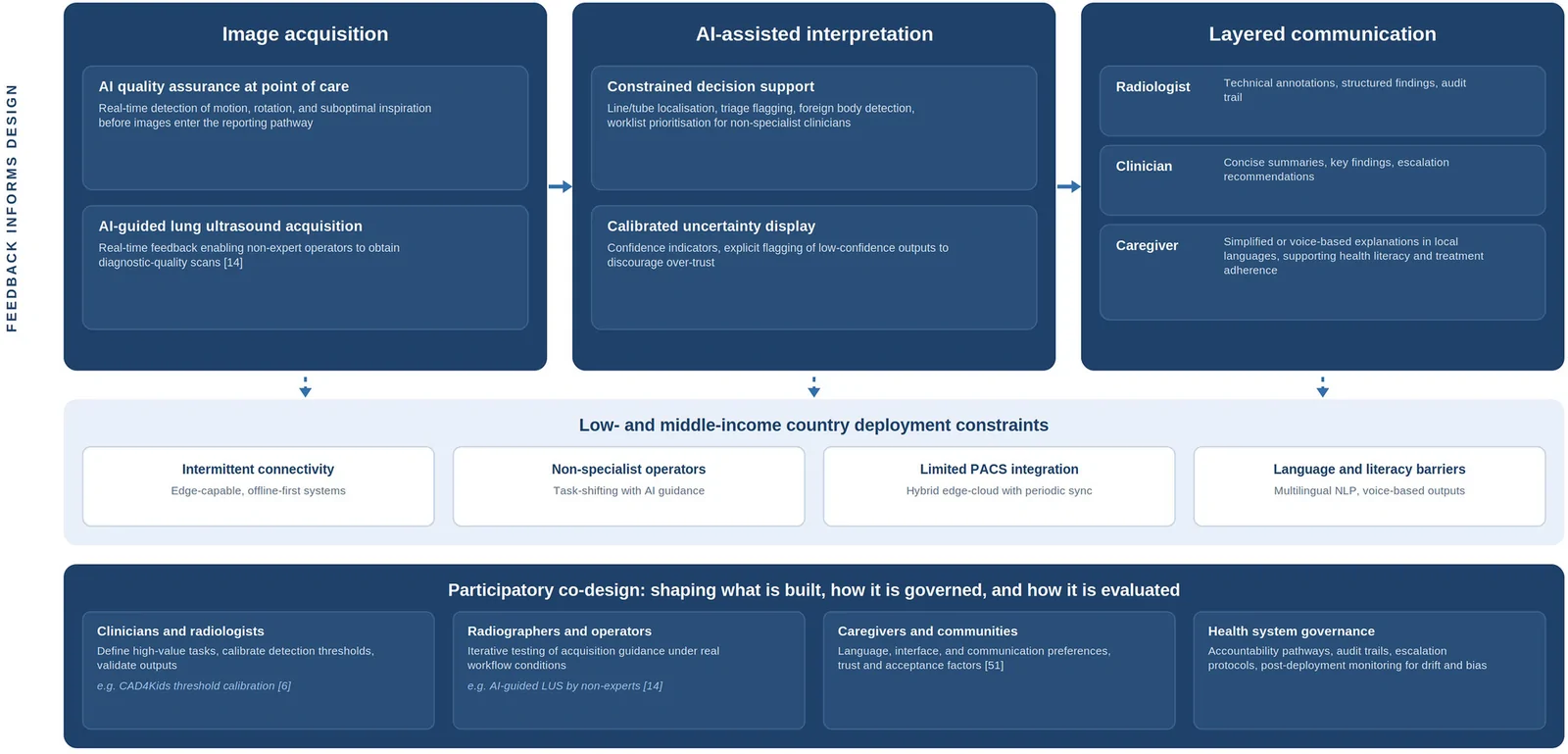
Proposed framework for practical integration of artificial intelligence in paediatric chest imaging in low- and middle-income country settings, illustrating the workflow from image acquisition through layered communication, the deployment constraints that shape implementation and the participatory co-design process involving clinicians, operators, caregivers and health system governance. *LUS*, lung ultrasound; *NLP*, natural language processing; *PACS*, picture archiving and communication system

These examples illustrate that participatory co-design is not merely aspirational: it is practically achievable and demonstrably improves both the relevance and the real-world performance of AI systems deployed in resource-constrained paediatric settings. Figure 3 provides a conceptual overview of how these elements connect across the clinical workflow, from image acquisition and AI-assisted interpretation through layered communication with clinicians and caregivers, within the deployment constraints and participatory processes that define safe implementation in resource-limited settings.

### Training AI-literate practitioners and strengthening accountability

As AI becomes more embedded in paediatric imaging workflows, radiologists will need skills beyond operating software. Training should emphasise domain shift awareness, uncertainty interpretation, automation bias and the practicalities of audit trails and monitoring, so that clinicians can act as responsible stewards of AI outputs rather than passive recipients [50]. This is particularly important in LMIC settings, where radiologists may be the primary interface between AI tools and broader clinical teams.

In parallel, ethical and legal accountability must be explicit. Paediatric patients are uniquely vulnerable to harm from missed diagnoses and biased outputs. Health systems therefore need clear policies on responsibility for AI-influenced decisions, documentation of AI outputs in the medical record, privacy and security safeguards, and defined escalation pathways when model behaviour is inconsistent with clinical expectations. Without these safeguards, “explainability” risks becoming cosmetic rather than protective.

## Conclusion

Paediatric chest imaging sits at the intersection of high clinical burden and high uncertainty in medical AI. Adult benchmark performance has not yet translated into reliable, equitable tools for children—particularly in LMIC contexts where tuberculosis, pneumonia and congenital cardiopulmonary disease remain major causes of morbidity and mortality.

Our experience with MedGemma and CAD4Kids illustrates two divergent trajectories: powerful generalist models that can fail on endemic paediatric disease and acquisition variability, and locally calibrated systems that can perform well within narrowly defined, clinically relevant tasks. Moving forward, the most defensible route is paediatric-first development: validation across developmental age bands and context-specific evaluation in the settings where children are imaged. AI must be explainable not only to clinicians but also communicable to caregivers, aligned to local languages and health literacy, and embedded within governance structures that preserve accountability.

The central question is no longer whether AI can interpret chest radiographs, but whether we design and deploy these systems in ways that genuinely benefit children. At present, most AI systems applied to paediatric chest imaging should be regarded as research tools or narrowly scoped clinical assistants rather than autonomous diagnostic systems—a distinction that must be preserved in both clinical practice and policy. The technology may be advancing quickly; paediatric safety, equity and real-world utility must advance faster.

## Supplementary information

Below is the link to the electronic supplementary material.

ESM 1 (DOCX 1.20 MB)

## Data Availability

No datasets were generated or analysed during the current study.

## Declarations

### Use of artificial intelligence

Artificial intelligence–assisted tools were used to support language editing, structural refinement and drafting of phrasing. All scientific statements, interpretations and conclusions were developed and verified by the authors using the cited literature and original clinical expertise. All AI-assisted suggestions were critically reviewed and edited, and the authors take full responsibility for the accuracy and integrity of the final manuscript.
