# Supplementary material for: Artificial intelligence in paediatric chest imaging: applications, challenges, and future directions
Source: Pediatr Radiol. 2026 Jun 3;56(9):1933–44. doi: 10.1007/s00247-026-06671-6 (PMC13546167; doi:10.1007/s00247-026-06671-6)
Supplement: Supplementary file 1 [file 247_2026_6671_MOESM1_ESM.docx]

**Supplementary Material 1.** MedGemma performance in adult pneumonia detection

A 35-year-old man presenting with fever and cough. Frontal chest radiograph shows a right lower lobe opacity consistent with pneumonia; MedGemma correctly identifies the abnormality, illustrating strong performance in familiar adult pathology when image quality is optimal. Case courtesy of Sorrentino S, Radiopaedia.org, rID: 15673, used under Creative Commons licence

**Supplementary Material 2.** Paediatric pulmonary tuberculosis case analysed using MedGemma 4B-it

A 10-year-old boy with pulmonary tuberculosis. **a** Frontal chest radiograph shows a left upper lobe cavity surrounded by consolidation; MedGemma reports a vague “left upper lobe opacity” without recognising cavitation or explicitly naming tuberculosis. **b** Frontal chest radiograph of the same patient with additional clinical context including HIV infection provided to the model; tuberculosis appears in the differential, demonstrating context-dependent performance. **c** Frontal chest radiograph with iterative prompting referencing classic tuberculosis radiographic signs, resulting in explicit mention of cavitation and tuberculosis in the output. **d** MedGemma output explaining tuberculosis findings and the importance of prolonged treatment to a caregiver in simplified language, illustrating potential multilingual natural language processing applications. Case courtesy of Laila Adel Mohsen, Radiopaedia.org, rID: 96737, used under Creative Commons licence

*HIV* human immunodeficiency virus, *TB* tuberculosis

**a**

**b**

**c**

**d**

**Supplementary Material 3.** Foreign body detection variability in paediatric imaging

A toddler (exact age and sex not reported) with an oesophageal foreign body. **a** Upright frontal chest radiograph with minimal rotation shows a coin in the upper oesophagus; MedGemma correctly identifies a metallic foreign body in the upper mediastinum. Case courtesy of Radiopaedia.org (anonymous contributor), used under Creative Commons licence. **b** A child (exact age and sex not reported) with an oesophageal foreign body. Supine, slightly rotated frontal chest radiograph shows a coin in the oesophagus, which MedGemma fails to detect, illustrating the model’s sensitivity to projectional and technical variation common in paediatric imaging. Image courtesy of Wikimedia Commons (user: Samir), CC BY-SA 3.0

**a**

**b**

**Supplementary Material 4.** Tuberculosis detection limitations in an adult patient from a low- and middle-income country

A 25-year-old woman presenting with cough. Frontal chest radiograph shows left upper lobe consolidation with densely calcified granulomas, radiographically suggestive of pulmonary tuberculosis confirmed on sputum microscopy and culture; despite these features and the clinical context of a high-burden setting, MedGemma 4B-it returns broad differentials including pneumonia, mass and fluid collection without explicitly naming tuberculosis, illustrating how models may under-prioritise endemic diseases in low- and middle-income country environments. Case courtesy of Gaillard F, Radiopaedia.org, rID: 12569, used under Creative Commons licence

*LMIC* low- and middle-income country, *TB* tuberculosis
